# Current Prospects for Plastic Waste Treatment

**DOI:** 10.3390/polym14153133

**Published:** 2022-07-31

**Authors:** Damayanti Damayanti, Desi Riana Saputri, David Septian Sumanto Marpaung, Fauzi Yusupandi, Andri Sanjaya, Yusril Mahendra Simbolon, Wulan Asmarani, Maria Ulfa, Ho-Shing Wu

**Affiliations:** 1 Department of Chemical Engineering and Materials Science, Yuan Ze University, 135 Yuan-Tung Road, Chung-Li, Taoyuan 32003, Taiwan; damayanti@tk.itera.ac.id; 2 Department of Chemical Engineering, Institut Teknologi Sumatera, Jl. Terusan Ryacudu, Way Huwi, Kec. Jati Agung, Lampung Selatan 35365, Indonesia; riana.saputri@tk.itera.ac.id (D.R.S.); fauzi.yusupandi@tk.itera.ac.id (F.Y.); andri.sanjaya@tk.itera.ac.id (A.S.); yusril.119280082@student.itera.ac.id (Y.M.S.); wulan.119280009@student.itera.ac.id (W.A.); maria.119280081@student.itera.ac.id (M.U.); 3 Department of Biosystems Engineering, Institut Teknologi Sumatera, Jl. Terusan Ryacudu, Way Huwi, Kec. Jati Agung, Lampung Selatan 35365, Indonesia; david.marpaung@tbs.itera.ac.id; 4 Graduate School of Biotechnology and Bioengineering, Yuan Ze University, 135 Yuan-Tung Road, Chung-Li, Taoyuan 32003, Taiwan

**Keywords:** polypropylene, polystyrene, polyvinyl chloride, high-density polyethylene, low-density polyethylene, polyurethanes, chemical–mechanical recycling

## Abstract

The excessive amount of global plastic produced over the past century, together with poor waste management, has raised concerns about environmental sustainability. Plastic recycling has become a practical approach for diminishing plastic waste and maintaining sustainability among plastic waste management methods. Chemical and mechanical recycling are the typical approaches to recycling plastic waste, with a simple process, low cost, environmentally friendly process, and potential profitability. Several plastic materials, such as polypropylene, polystyrene, polyvinyl chloride, high-density polyethylene, low-density polyethylene, and polyurethanes, can be recycled with chemical and mechanical recycling approaches. Nevertheless, due to plastic waste’s varying physical and chemical properties, plastic waste separation becomes a challenge. Hence, a reliable and effective plastic waste separation technology is critical for increasing plastic waste’s value and recycling rate. Integrating recycling and plastic waste separation technologies would be an efficient method for reducing the accumulation of environmental contaminants produced by plastic waste, especially in industrial uses. This review addresses recent advances in plastic waste recycling technology, mainly with chemical recycling. The article also discusses the current recycling technology for various plastic materials.

## 1. Introduction

Plastics are an integral component of our modern lives due to their wide range of applications in households and industry [1]. Worldwide plastic production is estimated at around 1.1 billion tons of plastic in 2050 [2]. The increase in interest in plastics as raw materials in various sectors comes from its ease of handling, transparency, and cost-effectiveness [3]. Plastics have shown extraordinary packaging performance for food, confectioneries, chemical products, and medicinal products [1]. Around 40% of plastic materials worldwide are used to store and package completed items from various factories. Nevertheless, massive plastic waste is generated, due to its mass consumption. Packaging is the most significant contributor to worldwide plastic waste, contributing to about 50% of the total weight [4]. Plastic waste from thermoplastic, thermoset, and elastomers of polymeric materials are not easily degraded [5] and could become abundant by producing primary environmental contamination. Moreover, the excessive amount of plastics generated over the last century, and poor waste management, have raised concerns about the depletion of fossil resources, the destruction of marine and terrestrial ecosystems, and climate change [6]. Therefore, the application of proper plastic waste management is critical to solving sustainability and environmental issues.

To date, plastic waste management has gained more attention worldwide due to its impact on human life sustainability. Typical plastic waste management strategies include landfills, incineration, microbial decomposition, thermal decomposition, mechanical pulverization, and recycling. Rapid and effective identification and classification of separate mixtures of waste plastic is challenging and this will be a crucial component in the waste plastic industry [7]. Therefore, plastic waste is mainly disposed of in landfills and discharged into the environment. These wastes, particularly plastic packaging, end up in rivers and seas, posing a significant hazard to aquatic habitats [5]. Landfill is becoming increasingly costly, as the volume of waste increases and landfill capacity decreases. More importantly, dumping plastic waste in a landfill could waste valuable resources and causes a series of problems, such as additive leaching and land occupation [8]. Meanwhile, incineration is commonly used in the energy recycling of plastic waste, since a significant amount of energy can be recovered, and the energy can be utilized to generate electricity, combined heat, power, or for other operations [9]. However, recycling waste plastics by incineration can be harmful, because various toxic components, which may cause carcinogenesis, teratogenesis, and mutagenesis, are detected in fly ash and residues, at concentrations exceeding the allowable limits [10]. Among these methods, plastic waste recycling simultaneously offers an acceptable and environmentally friendly approach.

Plastic waste recycling refers to the waste management process that collects plastic waste materials and turns them into raw materials reused to produce other valuable products. Recycling is not only a method for disposing of plastic waste, but it is also an effective process to minimize the need for virgin plastics, which can help lessen global warming [9]. According to the ASTM Standard D5033, plastic recycling can be categorized as primary, secondary, tertiary, and quaternary recycling [11]. Based on the mechanism of the methods, plastic waste recycling can be classified as mechanical, chemical, and biological recycling [5]. Chemical recycling, such as catalytic and thermal processes, can convert plastic waste into value-added chemicals/fuels. This process is a potential method to reduce plastic waste as a primary source of environmental issues. Due to the plastic manufacturing industry consuming almost 6% of all petroleum produced globally, extracting fuel oils from waste plastics can help reduce the global dependence on oil [12]. The activation energy of catalytic pyrolysis with the presence of a catalyst is decreased, and catalytic pyrolysis can be performed at a lower temperature, increasing the polymer conversion rate. The catalytic pyrolysis of waste plastics and petroleum sludge was performed. The catalysts include molecular sieves, transition metals, metal oxides, clays, and activated carbons used to recycle plastic, and molecular sieves and M-series catalyst (M = Al, Fe, Ca, Na, K) for treating petroleum sludge [13].

Mechanical recycling is often classified as primary or secondary recycling, and chemical and biological recycling is commonly classified as tertiary and quaternary recycling. Each method has its advantages and disadvantages, depending on the user needs. Another aspect required in the recycling of plastic waste is the separation of the different materials. For instance, PVC in the PET extrusion process damages the equipment, due to chlorine, decreasing product qualities, such as color and viscosity [14].

There are various separation methods for plastic waste recycling, including optical sorting (colors and peaks based), density separation (densities based), flotation (surface properties based), and Tribo electrostatic separation (effective surface work function based) [10]. Knowing the most acceptable combination of recycling and separation methods of plastic waste would be a powerful way to diminish the accumulation of pollutants in the environment caused by plastic waste.

This review aims to discuss the current technology of mechanical and chemical plastic waste recycling, to reduce plastic waste accumulation in the environment. Among several recycling methods, mechanical and chemical are typical approaches used for plastic waste recycling. Several research studies have developed mechanical and chemical plastic recycling methods to replace landfill and incineration methods. The chemical recycling of plastic waste depends on the degradation of the polymer chains. Meanwhile, mechanical recycling of plastic waste typically leads to re-granulation. Furthermore, this review focuses on the recycling method in general and the suitability of each recycling method for various types of plastic waste. In addition, the identification and separation methods of fresh plastic waste from the environment, until ready to be recycled, will also be discussed. The separation of various materials needs to occur before the actual recycling process. A better understanding of each plastic waste recycling method is necessary for policymakers to be able to determine the proper methods to solve the significant plastic waste issue.

## 2. Waste Plastic Recycling and Technology

The recycling process of plastic can be divided into various types: primary, secondary, tertiary, and quaternary recycling [15]. Primary recycling is the processing of a specified and uncontaminated material, commonly scrap, from an industrial process. Furthermore, to provide a good product quality, recycled scrap or waste plastics can be mixed with new materials [16]. Nevertheless, the primary recycling process needs homogeneous, clean, and non-degraded materials, such as packaging, bottles, and pre-consumer products, with the product of primary recycling being quite similar to a virgin one [17].

**Figure 1:**
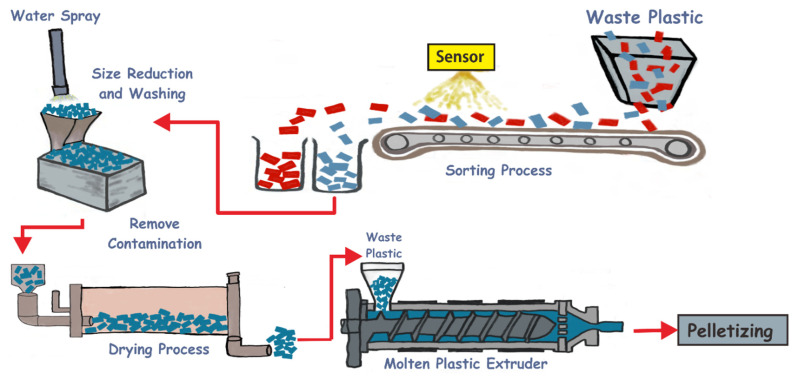
The technology of recycling waste plastic by a mechanical method: sorting process, size reduction, drying process, and molten plastic.

The mechanical recycling of plastic waste is secondary recycling; the most common method for recycling plastic waste. Mechanical recycling processes post-consumer plastics, to produce the raw materials for various plastic products [18]. In comparison, the recycling process depends on the chemical and physical properties of the waste plastic feed, in terms of its origin, composition, and form [19,20]. Figure 1 is related to the technology of recycling waste plastics by the mechanical method. Mechanical recycling includes several techniques, for instance, collection, separation, sorting, and washing [21]. The main objective of the waste plastic sorting process is to obtain high-quality recycled plastic goods, especially from a single polymer stream. Waste sorting technologies are based on various chemical-physical properties of the plastic, for instance, chemical compounds, size, color, and shape. Furthermore, the materials from post-consumer waste contain various polymeric materials and organic substances [17,22]. The subsequent process is size reduction. The typical process for size reduction involves cutting or shredding; nevertheless, this process depends on the type of plastic waste stream and plant layout. These processes may occur before or after the sorting stage [23].

The other processes include size reduction, extrusion, and granulation. These may occur in different sequences and at different times [19]. The extrusion and granulation processes are required to create a granulation that is possible to convert into flakes. Furthermore, the polymer flakes are typically loaded into an extruder, heated, and pressed through a die, to form a continuous solid polymer product (strand). This can be chilled in a water bath before the pelletized process. The granulation method is often utilized to convert the strands into pellets, which can then be used to produce new products [23]. To consider the full life cycle of polyurethane foams (PUFs), PUFs were upcycled and reshaped to bulk polyurethanes (PUs) using a transcarbamoylation reaction of up to five cycles. Moreover, four PUFs were prepared and reshaped by compression molding at 160 °C for 30 min, demonstrating the potential of this recycling pathway for PUFs from different origins [24].

**Figure 2:**
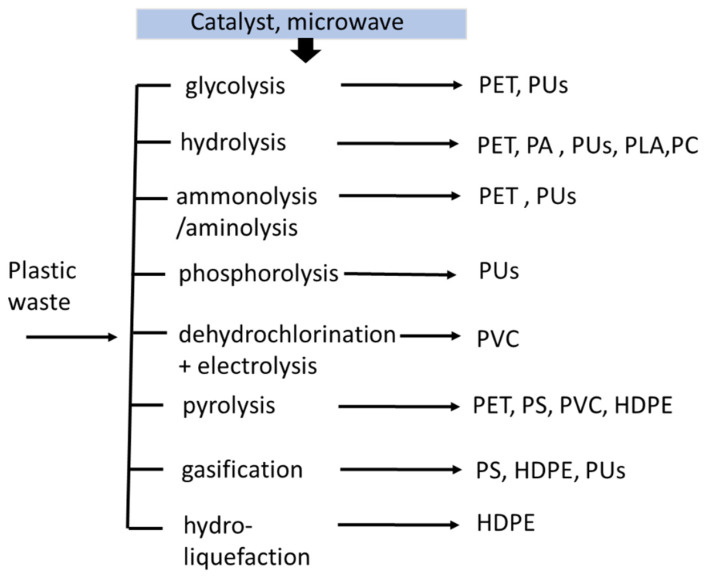
The chemical recycling route of plastic waste.

Tertiary or chemical recycling refers to the degradation of polymer bonds. As a result, the recovery of the oligomers monomers produces a smaller molecular weight. Hence, thermoplastic can be obtained with this method [25]. Some technologies can be applied in the following manner, as shown in Figure 2:

(a) Gasification: the polymer is utilized as a refuse-derived fuel using high temperature. It is converted to syngas with an H_2_/CO molar ratio of 2:1 in a gasifier; the syngas produced depend on the various polymers.
(b) Pyrolysis: the plastic waste is converted to pyrolytic oil, which is equivalent to diesel oil. In this chemical recycling, the calorific value of the polymer affects the energy content of the diesel [26].
(c) Glycolysis: the ethylene glycol and waste plastic are added in the presence of a catalyst. The long polymer chain is degraded into building blocks, which can be recycled to produce new polymers.
(d) Hydrolysis: when biopolymers (e.g., PLA) are heated and broken down to their monomer building blocks, they can be dissolved in water. These monomers can be recycled and utilized to make new products [27,28].

Quaternary recycling is a method of recovering energy using a combustion process of the waste polymer [29]. The plastic waste is incinerated. Nevertheless, the released energy is captured and replaced with heat and power. These strategies present a hierarchy of choice, in ascending order, from primary to quaternary, for managing resources and minimizing the processing costs of converters. Table 1 lists the advantages and drawbacks of various methods of recycling waste plastic. Plastic waste is commonly subjected to mechanical conversion in a closed-loop recycling method. Nevertheless, in metropolitan areas, this strategy cannot alleviate the accumulated plastic waste [30].

Currently, certain technologies have become the most used techniques for identifying and analyzing plastics. These techniques can be divided into elemental/atomic spectroscopies and molecular spectroscopies. Nevertheless, these methods utilize different types of physical phenomena [38]. Table 2 lists a summary of the sensor identification and sorting of waste plastic. The molecular spectroscopies produce information about the sample’s molecular identity and its molecular structure or conformation (the spectral signature of nearly any substance), allowing for accurate identification and characterization. Fourier transform infrared spectroscopy (FTIR), terahertz spectroscopy, Raman spectroscopy (RS), and near-infrared spectroscopy (NIR) are essential techniques in molecular spectroscopies. Fan et al. studied the admixture of microplastic containing polyethylene terephthalate (PET), polyethylene (PE), nylon (NY), polyvinyl chloride (PVC), and polypropylene (PP) by using Fourier transform infrared (FTIR) spectroscopy. The characteristic of wavelength numbers were PE (1472 and 1462 cm^−1^), PVC (712 cm^−1^), NY (3295 cm^−1^), PET (793 cm^−1^), and PP (841 cm^−1^) [39].

During transportation and storage of plastic cups and bottles used for mineral water packaging, the plastic particles can be released due to long-term exposure to light, heat, and unfavorable chemical environments. These types of plastic particles can be identified using quantitative surface-enhanced Raman spectroscopy (SERS) [40]. On the contrary, with FTIR, Raman spectroscopy is far more responsive to the molecular structure (electronic bonds) than the functional groups. Consequently, FTIR and RS have already been considered as complementary methods [38,41]. Molecules can be stimulated to a greater energy level when photons from a laser interact with the molecular vibrations. The majority of this energy will be dispersed via elastic scattering (or Rayleigh scattering), in which the energy of the released photons is equal to that of the laser photon. Raman spectroscopy determines the wavelength of inelastically scattered photons. The released photon has a higher or lower energy than the photon emitted by the laser, which can be seen as the spectrum of intensity over wavelength [42]. The chemometric characterization of Raman spectra was demonstrated to be successful for classifying PE with various densities, due to the intensities of CH_2_ with wagging and scissoring [43].

Laser-induced breakdown spectroscopy (LIBS) is a technology for elemental analysis, which has already been called “a future superstar” [44]. LIBS is already widely utilized for plastic sorting analyzers. The classification and identification of various types of waste plastics is the most common usage, consisting of different plastic objects; for instance, household applications, toys, electrical cables, containers, landmine casings, and various types of e-waste [45]. Furthermore, LIBS has received much attention for several plastic compounds such as plastic-based films, plastic-bonded explosives, and bio-plastic [46].

Several chemometric technologies can identify plastic polymers, and the data is collected using spectroscopic approaches. The broad category of chemometric technologies includes partial least squares regression (PLS), principal component analysis (PCA), and linear discrimination analysis (LDA) [42]. Henriksen et al. studied plastic identification using unsupervised machine learning models such as K-means clustering and PCA. In the thirteen types of plastics examined using PCA, the hyperspectral imaging with wavelengths ranging from 955 to 1700 nm proved that the spectral range was sufficient to identify plastics [47]. Furthermore, the most extensively used method of sorting waste plastic by multivariate analysis is with partial least squares discrimination analysis (PLS-DA), which is a very stable and straightforward approach for spectra data [48].

Near-infrared hyperspectral imaging (HIR-NIR) is a well-established technology for separating larger plastics in recycling plants and waste management. This sorting method is non-destructive, and the spectral range is a polymer fingerprint region with easily detectable C-N, N-H, and C-C absorption bands [49]. Vidal et al. investigated extensive microplastics identification using HIR-NIR. The microplastics had small particle sizes (<600 µm) and could be easily recognized, even though they were invisible by visual inspection or during handling. The features HIR-NIR are advantageous compared to traditional infrared (IR) spectrometers. A pixel size of 156 × 156 µm with a 75 cm^2^ scan area was probed in under 1 min. The specificity and sensitivity of waste plastics such as PE, PP, PA, PET, and PS were over 99% [50].

**Table 2:** Summary sensor for identification and sorting of waste plastic recycling.

Waste Plastics	Analyzer	Chemometric Tool	Wavenumber, nm	Accuracy, %	Ref.
Waste electrical and electronic equipment plastic (PP, PS, ABS, ABS/PC)	NIR512 by Ocean Optics	PLS-DA PCA-LDA	900–1700	99	[51]
Household waste (PE, PP)	Specim ImSpector N17	PLS-DA	1000–1700	100	[52]
Standard plastic samples (PE, PET, ABS, PS, PC, PP, and PVC)	NIR	PCA-SVM PCA-KNN PCA-ANN	900–1700	100	[53]
PS, PP, and ABS	RS	PCA-SVM	100–3300 cm^−1^	95	[54]
Waste plastics (PS, PP, PET, PVC, LDPE, HDPE)	MicroNIR	PLS-DA	900–1700	100	[55]
Black waste plastics (PS, PET, PP)	ATR FT-IR	FRBFNN	695–1376 cm^−1^	99	[56]
Black waste plastics (PS, PET, PP)	RS	FRBFNN	410–2871 cm^−1^	95	[56]
Waste plastics (PE, PP, PET, PVC, PS)	HSI-NIR	PLS-DA	1000–1700	100	[57]
Plastic solid waste (PET, PMMA, PP, PE, PS)	NIR	PCA-SVM	1000–1700	97.5	[58]
Black waste plastics (PET, PP, and PS)	RS	FRBFNN	200–3000 cm^−1^	95	[59]
Electronic household appliances (PP, ABS, PS)	RS	NA	1000 cm^−1^	94	[60]

### 2.1. Recycling Polypropylene

PP has a linear hydrocarbon chain, with a melting temperature of 160 °C [61]. PP is produced from propylene with a catalyst of metallocene or Ziegler-Natta. Furthermore, PP has excellent thermal, physical, and mechanical properties at ambient temperature. PP is an extensive application in plastics, stationery, furniture, food containers, and automotive industries [62]. PP recycling can be approached by chemical and mechanical methods, such as pyrolysis and hydrogenolysis. In addition, chemical recycling provides a chance to recycle waste plastic and convert it into higher value-added chemicals, especially for fuel additives. PP recycling approaches can be integrated with chemical refineries and generate a new generation of recyclable-by-design polymers [63,64].

**Figure 3:**
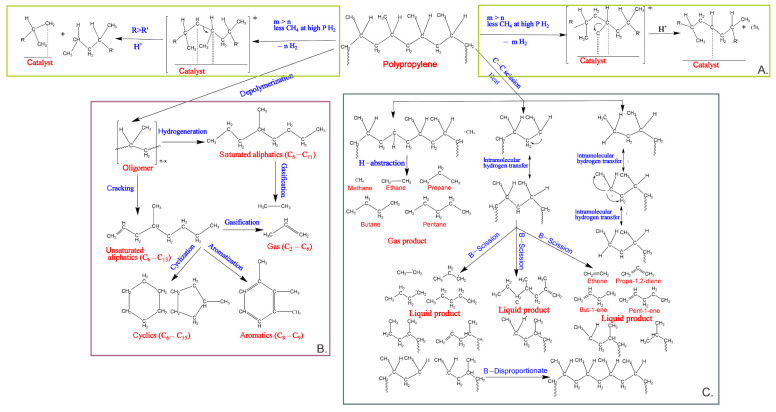
The depolymerization mechanism of polypropylene reaction: (**A**) hydrogenolysis into gas and liquid alkanes, (**B**) supercritical water depolymerization process, and (**C**) pyrolysis in a semi-batch reactor under atmospheric pressure [65,66,67].

Upcycling PP waste by hydrogenation has recently received much attention. Figure 3A illustrates the pathway of polypropylene degradation by hydrogenolysis. Chen et al. found a depolymerization mechanism of polypropylene under a catalyst by hydrogenolysis. The initial step of the reaction of hydrogenation is dehydration and adsorption. Hence, a hydrogen-depleted intermediate is formed. The hydrogenation of the fragments occurs during C-C cleavage, mainly resulting in new products. The lighter product with a R’-group will desorb more quickly [65].

Furthermore, Kane et al. investigated the detailed mechanism of depolymerization of waste polyolefin under hydrogenolysis. Generally, the hydrogenolysis process generates liquid alkanes over methane. There are several possible reactions during hydrogenolysis: (1) σ-Bonds metathesis is produced by primary carbons (1–3 kcal mol^−1^); (2) the long-chain moves into β-H by an elimination process and reduces the yield of E-alkenes, with substitution by Z-alkenes, (3) the elimination of β-Alkyl and methyl (1 kcal mol^−1^) following the majority Zr–C bond formation (2 kcal mol^−1^); and (4) the stabilization process, by eliminating β-alky. A catalyst can promote the removal of the β-alkyl compound, degrading the long chain of PP more easily than LDPE [68]. The upcycling of plastics becomes exothermic via hydrogenolysis/hydrocracking, decreasing the reaction temperature to around 300 °C [69]. Noble metal nanoparticles placed on silica, alumina, ceria, or carbon in the presence of hydrogen can easily break C–C bonds in the polyolefin backbone through hydrogenolysis, resulting in low molecular-weight wax or small molecules of hydrocarbons [70,71]. Rorrer et al. investigated the hydrogenolysis of PP, and the process was under mild temperature (225–250 °C), with a selected hydrogen pressure (20–50 bar). In addition, the ruthenium nanoparticle supported on carbon (5 wt% Ru/C) was effective as a heterogeneous catalyst for the degradation of PP via hydrogenolysis. The hydrogenolysis of PP produces a liquid product with a yield of liquid over 68%, with the range of chemical compounds being liquid (C_5_–C_32_) and gas (C_1_–C_5_) [72].

The depolymerization of PP waste with supercritical water for liquefaction is illustrated in Figure 3B. In the first stage, PP waste degrades into oligomers in a short time (<0.5 h). Then, the bulk of unsaturated aliphatic is changed into cyclic via cyclization, when the reaction time is increased slightly. Most unsaturated aliphatic is converted into cyclic by cyclization. At the same time, small amounts of unsaturated aliphatic (olefin) may become saturated aliphatic (paraffin) and aromatics. Aromatization can theoretically occur through cyclic dehydrogenation or unsaturated aliphatic cyclotrimerization (olefins) [73]. Degradation of polymers takes place in the viscous polymer phase during pyrolysis. On the other hand, the supercritical water for liquefaction is produced by partial dissolution of the molten polymer phase. Furthermore, the dissolution of the polymer phase enhances unimolecular the reactions and polymer dissociation, for instance, β-scission. Consequently, coke formation, polycondensation, and gas creation processes are prevented [74].

Pyrolysis is a thermochemical degradation process; it can degrade the polymer compound at high temperatures without oxygen [75,76,77]. Non–catalytic pyrolysis is a standard technique for recycling large molecules of PP. Nevertheless, it requires a high temperature of up to (573–1173 K) and a long reaction time, and it produces a wide range of chemicals, for instance, alkanes, alkenes, aromatics, and gases [78]. Figure 3C shows the possible reaction during pyrolysis of PP. The mechanism of PP by pyrolysis starts with chain fission, radical recombination, allyl chain fission, intermolecular hydrogen abstraction, midchain β-scission, disproportionation, 1,3-end-hydrogen transfer, 1,4-end-hydrogen transfer, 1,5-end-hydrogen transfer, 1,6-end-hydrogen transfer, 1,3-mid-hydrogen transfer, 1,4-mid-hydrogen transfer, and 1,5-mid-hydrogen transfer [79]. Singh et al. studied waste tube tires (WTT) and waste polypropylene (WPP) for conversion into diesel fuel via catalytic pyrolysis and base SrCO_3_. The major products were aromatics, naphthenes, monohydric alcohols, esters, amides, and halides. Diesel fuel from WTT and WPP has research octane numbers of 89.65 and 87.32, respectively [80].

Catalysts are frequently added to minimize the reaction time and increase product distribution. Several catalysts have shown increased yield products in PP processing with certain reactor conditions (Table 3). Acid catalysts, such as FCC catalyst, mesoporous silica, and zeolites, have been used in PP depolymerization [81,82]. The catalytic pyrolysis of medical waste PP under CO_2_ was studied using Ni/SiO_2_ catalyst. The chemicals, saturated hydrocarbons, olefins, and alcohols were produced in greater quantities when the H_2_/CO ratio was controlled. Nevertheless, the catalytic pyrolysis process produced hydrocarbons (≥C_2_) and CH_4_ and H_2_ [83]. The depolymerization of PP pyrolysis was carried out in a stainless-steel batch reactor by Dutta et al.; the reaction temperature was 470 °C, with a maximum yield of 65%, and with the absence of a catalyst. On the other hand, a silica-alumina catalyst was added to the pyrolysis process. The maximum yield of liquid product was up to 75%, due to silica-alumina having better porosity and acidic properties. The caloric value of the liquid product was 41.1 MJ/Kg, with the major products being benzene, toluene, and xylene [84]. Harmon et al. studied the kinetic depolymerization of PP, and there were various methods used to investigate the activation energy (Ea), such as the Kissinger and Friedman methods. The Ea with the Friedman method was 235 kJ/mol, with a range of conversion up to 30–70%. Moreover, Ea = 236 kJ/mol and A = 5.60 × 1014 s^−1^ were determined by the Kissinger methods. The reaction profile was only 63% as wide as a first-order reaction [85].

Compared to pyrolysis, the gasification process is conducted at higher temperatures, with a range of 700–1000 °C. The gasification agents, such as CO_2_, air, O_2_, and steam, are added to partially oxidize carbonaceous materials, producing a syngas consisting of CO, H_2_, CO_2_, and CH_4_. Small amounts of other hydrocarbons can also be found. Syngas of high quality is needed for both chemical synthesis and fuel. Gasification with O_2_-enriched air can generate syngas with a high calorific value and gas concentration in term of minimizing the N_2_ dilution. Therefore, the air separation process can be expensive. To produce pure oxygen for partial oxidation, gasification can be combined with chemical looping processes and using transition metal-based oxygen carriers instead of gaseous O_2_ [97,98]. Xiao et al. investigated the recycling PP plastic by air gasification in a fluidized bed gasifier, to produce a low tar content and fuel gas with a calorific value of 5.2–11.4 MJ/Nm^3^. Approximately 250 mg/N m^3^ of tar was found in the gas products, with a yield of fuel gas up to 3.9 N m^3^/kg [99]. In addition, the dissolution process is one of the methods to depolymerize large compounds of PP into small molecules. The dissolution process can be affected by molecular weight, polymer size, dissolution time, temperature, and concentration [100]. During polymer breakdown, mechanisms such as solvent diffusion and chain disentanglement are implicated thermodynamically. Furthermore, polymer self-diffusion is critical during chain disentanglement [101].

### 2.2. Recycling Polystyrene

PS is an aromatic polymer produced by polymerizing a styrene monomer. PS is a popular material because it has excellent physical properties, such as strength, durability, versatility, and low cost [102]. PS is extensively used in the form of expanded PS foam, which has a low thermal conductivity, good resistance to many corrosives, and is nearly impervious to moisture [103]. Styrene polymerization can be achieved through various intermediates and/or active species, such as cationic, coordination polymerization, anionic, and radicals. Free-radical polymerization is mostly used in the commercial production of atactic PS with a higher molecular weight, up to 200,000–300,000 g/mol. It can produce an amorphous polymer with a comparatively high glass transition temperature of Tg = ~100 °C [104].

**Figure 4:**
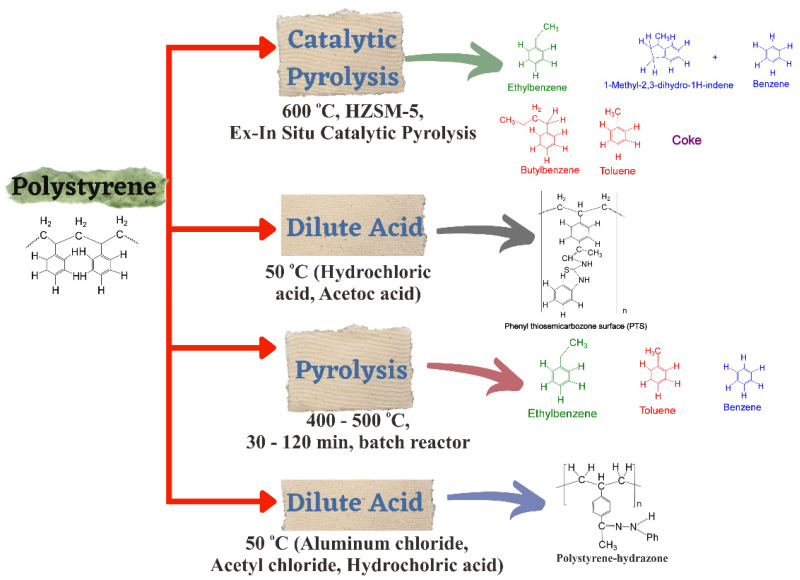
Scheme of valuable liquid products for PS depolymerization for different reaction paths.

PS is difficult to degrade in natural environments. Nevertheless, chemical, mechanical, and thermal recycling are used to degrade the large molecule of PS. One of the methods for chemical recycling is dissolution. Figure 4 illustrates PS degradation by dissolution and a scheme of liquid products valuable in PS depolymerization, such as catalytic pyrolysis, dilute acid, and pyrolysis [105,106]. The polymeric components are first dissolved, then various processes are used to recover the solvent and polymer [107]. The recycling of foamed polymers by using solvents has several advantages. Filtration can be used to eliminate any insoluble impurities, leaving the polymer clean for any further treatment. Additionally, the dissolution process enables the separation of plastics from other waste and insoluble polymers, according to their chemical structure, a process known as selective dissolution. For expanded materials, dissolving the foam in a suitable solvent results in a significant volume decrease (over 100 times), lowering transportation expenses [108].

Cymene, terpinene, phellandrene, and limonene are used for recycling expanded polystyrene. Furthermore, limonene is an excellent solvent and antioxidant for recycling expanded polystyrene throughout the heating process. A prior work discovered that using d-limonene as a diene compound in the thiolene reaction dissolves PS, allowing it to be recovered from the solution [101]. Furthermore, p-xylene/n-heptane, methyl ethyl ketone (MEK)/methanol, and MEK/n-hexane are suitable for recycling PS foam. Conventional solvents such as methanol/xylene can dissolve PS at various temperatures. Within a specific temperature range, a rising temperature will contribute to the rapid recovery of PS. Due to environmental damage, a more environmentally-friendly solvent (D-limonene, produced from citrus fruit rinds) was utilized to obtain 100% recycling of expanded polystyrene [109]. Gil-Jasso studied the application of essential oils to dissolve and recover PS waste. Various solvents were applied, such as chamomile, thyme, star anise, and eucalyptus oil, with a total percentage of PS recovery of more than 95%, and with a reaction time up to 833 s [110]. Polarity influences the solubility of polymers in solvents. A polymer is naturally inclined to dissolve more readily in the non-polar solvents that are chemically and physically closest to the XPS. Nevertheless, polar solvents can also be utilized in the recycling process if they do not have a strong tendency for hydrogen bond formation [108].

Pyrolysis and catalytic degradation of PS is a waste treatment process (thermal recycling) that can be utilized as a substitute for landfill disposal [111]. Simple thermal cracking at low temperatures can convert PS to styrene, without catalysts. PS pyrolysis is primarily influenced by catalyst presence, reaction time, temperature, and reactor type. Products consist primarily of liquid chemicals at low temperatures (mono aromatic). Coke and gas will be increased slightly at higher temperatures, and the liquid fraction includes many aromatics (dimer, trimer) [112]. Furthermore, adding a catalyst reduces the residence time of polymer degradation in the reactor and decreases the process temperature, by lowering the activation energy by breaking the chain of C–C bonds. PS catalytic depolymerization can be split into acid and alkaline [113]. Numerous research works on the catalytic pyrolysis of PS have been performed, including metallic oxides (alumina, alumina-silica, CuO/Al_2_O_3_, BaO, Al_2_O_3_, SiO_2_, K_2_O, CaO, or silica), assisted transition metals, mesoporous materials (K_2_O−BaO/MCM−4, K_2_O/Si−MCM−41, MCM−41 sepiolite derived from nature), as well as clay (pyrophyllite, albite, halloysite, montmorillonite) [112,114].

The degradation of PS waste with various basic and acidic catalysts was studied by Anwar et al. The catalytic pyrolysis was conducted with calcium oxide at temperatures ranging from 300 to 350 °C and at atmospheric pressure. The total distillate recovery was up to 77%. On the other hand, a metal carbonate catalyst generated pure styrene. Nevertheless, the yield of styrene was low [115]. The mechanism of PS catalytic pyrolysis with montmorillonite and albite as a catalyst was investigated before. The first stage of the reaction process was β-scission, followed by intermolecular H transfer, with major products being ethylbenzene and styrene [114].

Zayoud et al. studied pilot-scale pyrolysis of PS via a CSTR reactor and extruder under vacuum conditions. In the temperature operation increased to become 450 and 550 °C, the styrene yield rose 36 and 56%, respectively. In addition, at 450 °C and 0.02 bar, the yield of benzene, toluene, ethylbenzene, and xylene was enhanced from 4 wt% to 17 wt% at 450 °C and 1.0 bar [116]. Furthermore, Amjad et al. investigated PS catalytic cracking with Nb_2_O_5_ and NiO/Nb_2_O_5_ as catalysts. The Nb_2_O_5_ catalyst showed the highest catalytic cracking activity under a semi-batch reactor at 400 °C. The yield of ethylbenzene, toluene, α-methyl styrene, styrene, and dimers was 6%, 4%, 13%, 50%, and 6%, respectively [117]. PS thermal depolymerization can be applied to produce a styrene monomer. Nevertheless, this method has certain drawbacks, including equipment blockages and high-temperature requirements [113].

A microwave can be utilized in the pyrolysis process of PS. Microwave radiation interacts well with PS waste via the medium’s dielectric constant and generates a quick heating process. Microwave processing features contain challenging characteristics: (a) rapid heating, (b) reduced energy consumption, (c) poor thermal inertia, and (d) high conversion efficiency of power [118]. The reaction was made possible by adjusting the microwave, temperature, powder, microwave design, catalyst, and absorber. The optimal temperature range for liquid products is between 600–500 °C, whereas a temperature beyond 700 °C produces more gas products. Silicon carbide and carbon are common absorbers used to increase microwave absorption. The synergistic interaction between reaction time, catalyst, and temperature enables the breakdown of long-chain hydrocarbon molecules [119]. PS pyrolysis through the microwave–metal interaction was studied by Hussain et al. To induce rapid pyrolysis at elevated temperatures, an iron mesh of different shapes (cylindrical mesh, strips, and iron cylinder) was added. The cylindrical mesh produced heat within the temperature range of 1100–1200 °C and had a higher conversion, with a total liquid, solid, and gas of 80%, 5%, and 15%, respectively [120]. Rex et al. investigated the pyrolysis of a mixture of PS and polypropylene using a microwave-assisted method, with various types of activated carbon biomass. The operating condition of microwave pyrolysis was 900 W, with a reaction time of 10 min. The polymer and absorbent ratio was 10:1, and the maximum oil yield was 84.3 wt% [121].

### 2.3. Recycling of Polyvinyl Chloride

**Figure 5:**
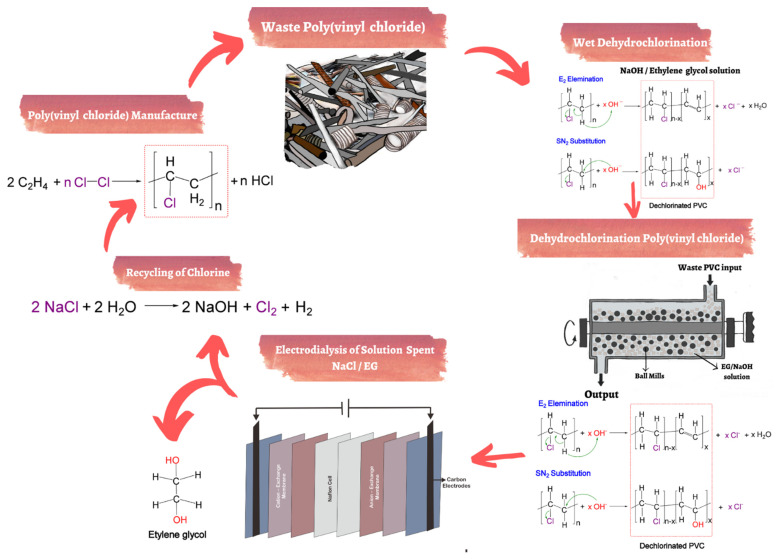
Technology for recycling of PVC waste by dehydrochlorination and electrodialysis [131].

PVC is widely used in extensive applications, such as packaging, construction, electronic industries, and automotive products. Furthermore, PVC has excellent electrical, thermal, mechanical, and chemical resistance properties [122]. PVC can be degraded with a lower temperature reaction than other plastics. The detailed mechanism of PVC breakdown has been investigated with various models, which were modeled as three processes: (1) converse PVC through several intermediates compounds and HCl; (2) intermediate compounds are degraded into volatile compounds and polyene chains; and (3) polyene breakdown into toluene (and also other aromatics) [105]. Figure 5 shows how dehydrochlorination and electrodialysis are applied to recycle PVC waste. The recycling of PVC is divided into several parts. The dechlorinating process starts with a mixture of PVC waste and NaOH/ethylene glycol. The PVC waste is dechlorinated and transferred to the EG solution in the form of Cl. Then, the next process is NaCl recovery by electrodialysis with EG solution containing Cl^−^ and Na^+^ through cation and anion exchange membranes [123]. Furthermore, Kameda et al. studied the electrodialysis of a NaCl/EG solution mixture through ion-exchange membranes. After 5 h, a high desalting ratio was obtained up to 98%. Nevertheless, the Donan effect was decreased by 0.5 wt.% of the efficiency NaCl, with total voltages greater than 4 V [123].

The other process for recycling PVC is pyrolysis. Nevertheless, PVC pyrolysis has some issues, because this process can produce fuel oil containing a large amount of chlorine [124]. The Cl^−^ in fuel oil products of pyrolysis can cause serious corrosion to parts of the machine and transfer toxic chemicals into the environment. As a result, the dichlorination process should be conducted before converting PVC into a high-quality fuel via pyrolysis [125]. On the other hand, catalytic and noncatalytic pyrolytic processes are used for PVC waste. Catalytic pyrolysis adds a catalyst, to increase the dichlorination process, while adding a sorbent reduces the product’s chlorine compound.

Zakharyan et al. reported treatments of virgin and mixture PVC (multicomponent and binary PVC mixture, chlorine- and bromine-compounds mixtures, biomass, and municipal plastics waste) [126]. Pan et al. studied chlorine transformation and migration during PVC pyrolysis using TG-FTIR-MS methods. This showed that pyrolysis occurs in two primary steps: the first step of the reaction is using a temperature between 200–360 °C. This phase includes the dichlorination of PVC, which produces a massive amount of benzene and hydrogen chloride. The second step is a reaction temperature of 360–550 °C. The polyethylene chain is broken in the second step, due to a large amount of aromatic organic substances and chlorine-containing compounds [127]. In addition, the flash pyrolysis of PVC was conducted with a temperature reaction up to 500 °C. The major products were HCl, alkenes, monocyclic aromatics, and PAHs at 3.02%, 2.86%, 33.5%, and 48.3%, respectively [128].

Furthermore, the thermodynamic and kinetic parameters of a PVC cable sheath were investigated by Liu et al. The range of activation energy in the first stage was 132–149 kJ/mol, with the average activation energy being 141 kJ/mol. On the other hand, the activation energy increased in the second stage to 193.8–266.4 kJ/mol, and the median activation energy was 235.3 kJ/mol using the Flynn–Wall–Ozawa method [129]. Zhou et al. studied the upcycling of PVC waste into carbon compounds, chlorides, and pyrolysis gas using a one-pot dichlorination–carbonization-modification approach. The total solid yield of dechlorinated PVC was up to 80.8 wt% at 700 °C [130].

Hydrothermal treatment is an effective method for removing chlorine from PVC [125]. A hydrothermal supercritical water treatment was evaluated to depolymerize PVC waste into organic compounds, such as gas and liquid products. The chlorine atoms were dissolved in water and did not cause the formation of organochlorine compounds. The sequential occurrence of three reactions was then postulated as a mechanism, as follows: (1) the zipper dehydrochlorination method was used to remove HCl from PVC, causing conjugate double bonds to form in the polymer chain; (2) polymer chain breakage; (3) aromatic molecules were synthesized by combining broken chains [132]. Enomoto et al. studied PVC depolymerization under high pressure in hot water [133]. Hydrothermal depolymerization under supercritical and subcritical regions was investigated by Takeshita et al. After the 300 °C degradation process, the chlorine compound in PVC was dissolved in water, and hazardous chlorinated organic compounds were detected in the gas and liquid fractions. The primary product with a temperature reaction of 250–350 °C was polyene as a residual solid and an aliphatic–aromatic compound in the gas and liquid fractions [132]. Zhao et al. studied the hydrothermal dichlorination of PVC wastes with an alkaline additive. Numerous chemical compounds were added, such as NH_3_H_2_O, KOH, Na_2_CO_3_, NaHCO_3_, and NaOH, which took place in subcritical Ni_2_^+^ and involved water at 220 °C for 30 min. In addition, Na_2_CO_3_ is the most promising additive, due to its high dichlorination efficiency up to 65.1% [134].

### 2.4. Recycling of High-Density Polyethylene and Low-Density Polyethylene

High-density polyethylene (HDPE) and low-density polyethylene (LDPE) are the most popular forms of polyethylene. The manufacturing of HDPE adds the organometallic catalyst to polymerize ethylene. HDPE contains a higher proportion of crystalline regions than LDPE and is, hence, opaque and harder. The polymer chain in HDPE can be 500,000 to 1,000,000 carbon units long, with little branching. HDPE offers a wide range of applications in numerous plastic products, such as food containers, cleaning products, pipes, cables, tubes, and thin-film coating [135]. The recycling of HDPE waste is conducted using fluid catalytic cracking, pyrolysis, and gasification; it follows the open-loop recycling of HDPE waste [136].

**Figure 6:**
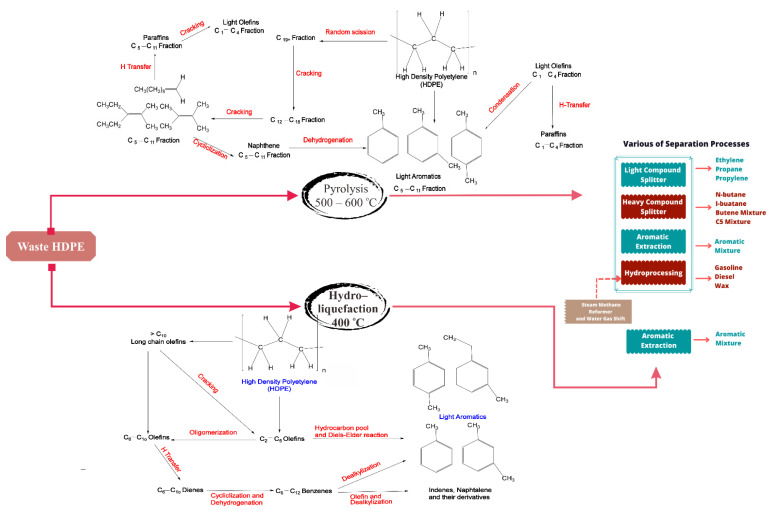
Possible routes of using depolymerized HDPE waste for aromatic hydrocarbon formation (**top**) by fast pyrolysis, using FCC spent catalysts in a fountain-confined conical spouted bed reactor, (**bottom**) by hydro-liquefaction over Ni/HZSM-5 [157,158,159].

The possible routes of using depolymerized HDPE waste for aromatic hydrocarbon formation are shown in Figure 6. The pyrolysis pattern of HDPE and LDPE is more intricate, and obtaining a large ethylene yield is challenging. The use of catalysts is an attractive solution to generate the desired product, especially with ethylene as the monomer of polyethylene. This process is called catalytic cracking. It can reduce energy consumption, due to lower temperatures than in thermal pyrolysis [137,138]. Singh studied the high yield liquid product of waste polyolefins pyrolysis, with a result of up to 92%, by applying MgCO_3_ as a catalyst, with the primary products being aliphatic, alcohol hydrocarbons, ester, acetate, and aromatic [139]. Furthermore, Zeolite, e.g., HZSM-5, is frequently utilized in major catalytic reactions, due to its larger specific area, high selectivity, pore structure, and acid group, which provides a hydrogen transfer reaction [140,141]. Integration of a fluidized bed reactor filled with HZSM-5 catalysts for the pyrolysis process, and pressure swing adsorption (PSA) for light components, as well as an inert gas, such as nitrogen separation, is one of the current technologies for polyethylene waste recycling process. Hernandez et al. found that the optimum temperature for FBR, and which produced high-yield gaseous compounds, was 500 °C [142]. The presence of HZSM-5 favors C3–C5 hydrocarbons in large proportions. Meanwhile, Hernandez et al. investigated the HUSY catalyst with a lower ratio of silica/alumina and a larger surface area than the HZSM-5 catalyst. The results showed that C_5+_ components were the primary product. The gaseous compounds from the pyrolysis reactor were condensed to split light and heavy components in the separator [143].

Five distillation columns were used in light component separation to obtain ethylene, propane, and propylene. In the first distillation column, known as a demethanizer, methane was removed at 6 °C and 20 bar. The remaining light compounds were the bottom product of the demethanizer, and they then entered a second fractionation called a deethanizer, to split C_2_ (ethane and ethylene) and C_3+_ as the bottom product [144]. Pure ethylene was obtained from the C_2_ stream entering to deethylenizer, and a third distillation column was used with an operating temperature and pressure of −26 °C and 20 bar, respectively. The bottom product from the deethanizer was distilled in the fourth fractionation to produce C_3_ (propane and propylene) and C_4+_ products mixed with heavy components from the separator in the pyrolysis unit. The last distillation yielded high propylene purity in the top column and propane in the bottom column [145].

Second step C_4_ separation, is a technology used to recover n-butane, i-butane, butene mixture, and C_5_ mixture. N-butane and i-butane are the raw materials for liquid petroleum gas (LPG) production, while butene mixture (trans-butene, 1-butene, isobutene, cis-2-butene, and 1,3-butadiene) and C_5_ mixture (n-pentane and i-pentane) are frequently used as copolymers and solvents [143,146]. A mixture of heavy components from a light separation and pyrolysis unit was heated and split in a flash drum to remove the liquid aromatic mixture. The top product of the flash drum was condensed before entering the first fractionation, called the C_4_ splitter. The column’s overhead liquid was sent to the n-butane separator (4 bar, 35 °C), where the n-butane product was placed in the bottom column. The liquid in the top n-butane column contained an i-butane and butene mixture, refined in the butene column (4 bar, 35 °C), whereas the overhead liquid was an i-butane stream at the bottom column, producing a butene mixture. Moreover, the bottom product of the C_4_ splitter flowed, to the C_5_ splitter to generate a C_5_ mixture in the top section and the rest of the heavier hydrocarbon was in the bottom column, which was a mixed aromatic mixture stream from the flash drum [147,148].

Aromatic components contain a mixture of benzene, toluene, and xylene (BTX), compounds that are regularly utilized as a feedstock in chemical industries. Liquid–liquid extraction is a popular method for recovering aromatic mixtures. There are many types of solvents, such as triethylene glycol (TEG), sulfolane, N-formylmorpholine (NFM), and N -methyl-2-pyrrolidinone (NMP), utilized to extract aromatic compounds [149,150]. The sulfolane process patented by UOP is commonly used in commercial plants, due to its high selectivity and boiling point, but low dissolvability. Therefore, to solve these drawbacks, a mixture of two solvents is the best option to increase the selectivity and lower the recycling rate and ratio of extractant [151,152]. Extraction of aromatic compounds using a co-solvent of TEG and sulfolane was studied by Galie et al. The significant selectivity of xylene was increased.

Meanwhile, the solvent and recycle feed ratios were reduced by 20% [151]. Other works showed that adding mixed solvents of sulfolane-NMP could extract 99% of benzene from reformate, and the distillate could be directly utilized as automobile gasoline [153]. However, liquid extraction process units have some drawbacks, due to their high investment cost. Conventional distillation is widely used for purifying the mixture of the components. Nevertheless, due to binary azeotrope conditions, the aromatic mixtures cannot be separated by traditional distillation. Extractive distillation (ED) is an alternative method to extract aromatic hydrocarbons with a high energy efficiency, low equipment investment, and modest process units. ED requires a particular component to raise the volatility to near boiling point [154,155].

The solvent in liquid–liquid extraction can be applied as a third compound in ED. Wang et al. reported that co-solvents of NMP and sulfolane were used to extract aromatic components with the ED technique. A feed with aromatic and non-aromatic substances flowed to the ED column (2.5 bar, 120 °C). A non-aromatic product was fed to the rectifying column in the top column, to produce non-aromatic compounds, and solvents were carried over to the overhead ED column. The bottom stream of the ED column entered the solvent recovery and regeneration column (1.01 bar, 101.4 °C), to obtain a high purity of aromatic mixtures and regenerated solvents, to reuse in the ED column. The recovery of aromatics with the ED method reached 99.92% [156].

On the other hand, adding hydrogen compounds is required to convert non-aromatic hydrocarbons into valuable products in a hydrogenator. Steam methane reforming, combined with a water gas shift reaction, is a mature process to produce hydrogen. A methane feed is mixed with steam, to carry out a methane reforming reaction. The products are hydrogen, carbon dioxide, carbon monoxide, and unconverted methane [160]. In addition, the water gas shift process is needed to boost hydrogen levels. Then, a PSA is installed to purify hydrogen from carbon dioxide, carbon monoxide, and unconverted methane. Afterward, the high-purity hydrogen and non-aromatic hydrocarbons are first heated to 200–450 °C, before flowing into the hydrogenation reactor. Gasoline (C_6_–C_7_), diesel (C_8_–C_16_), and wax (>C_16_) are produced in a hydrogenator using NiMo catalyst and can be applied as a fuel and chemicals [161,162].

Table 4 shows the product distribution of catalytic pyrolysis of LDPE and HDPE waste. In the catalytic pyrolysis of PE waste, a critical issue is catalyst deactivation, which occurs when many tiny molecules enter the pores of the catalyst, and macromolecular hydrocarbons that plug the pores are formed. Consequently, the lifetime of the catalyst is short [163]. A hydro-liquefaction process combining cracking and hydrogenation is proposed as an alternative technique to convert PE waste directly to liquid fuels or aromatic compounds. In a hydro-liquefaction reactor, Ding et al. tested three catalysts (Ni/HSiAl, NiMo/HSiAl, and KC-2600) to convert HDPE waste to liquid fuels. The results showed that Ni/HSiAl produced high yields of light hydrocarbons (≤C_13_) and the properties of the product were better than commercial gasoline, owing to a lower content of aromatics and high isoparaffin components [164].

Furthermore, Pan et al. investigated aromatic production from HDPE waste via the hydro-liquefaction method, using HZSM-5 with Ni to stabilize the aromatic product. Xylene was the dominant aromatic product, with a maximum yield of 28.9% at 400 °C. The aromatic selectivity reached 64.8%, with reaction time up to 4 h and a loading of Ni up to 15 wt% [158]. The patterning process of hydro-liquefaction is typical of direct coal liquefaction. Therefore, constructing a hydro-liquefaction plant can implement a straightforward coal liquefaction process.

### 2.5. Recycling of Polyurethanes Waste

**Figure 7:**
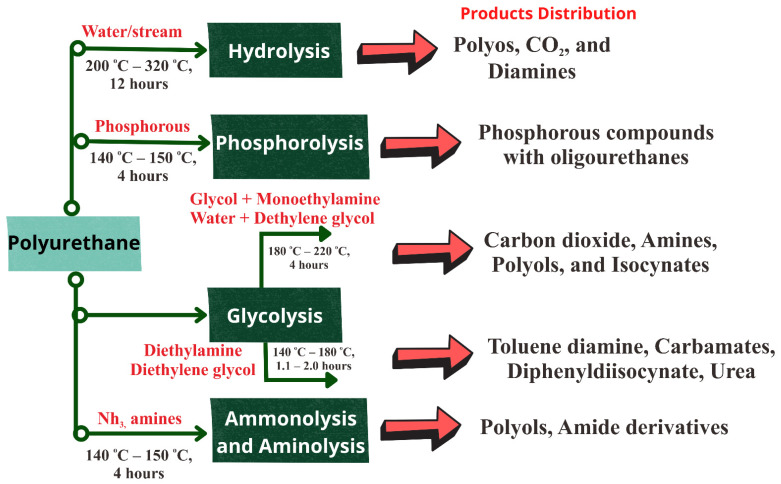
Alternative depolymerization routes for polyurethane waste [173].

Polyurethanes (PUs) are essential materials, due to the thermoset and thermoplastic that can modify their chemical, thermal, and mechanical properties by reacting with polyisocyanates and polyols. The major polymers with urethane groups (–HN–COO–) are classified as PUs, regardless of the rest of the molecule [170]. In addition, the polyether polyols based on polyethylene oxide, PP, aliphatic polyester polyols, tetrahydrofuran, polycarbonate polyols, aromatic polyester polyols, polybutadiene polyols, and acrylic polyols are the most often used polyols in the manufacturing of PUs [171]. The thermochemical recycling of PUs includes alcoholysis, glycolysis, ammonolysis, and hydrolysis [172]. Figure 7 shows the alternative depolymerization routes for polyurethane waste.

Furthermore, numerous studies have considered recycling PUs by hydrolysis. The recycling products of PUs are a high-quality yield of polyol, isomeric toluene diamines, and CO_2_. This was achieved by dry atmospheric pressure steam under a temperature range of 190–230 °C [173]. Nevertheless, urethane linkages are quite stable, and protective groups such as the benzoxycarbony group are often utilized to cover their amino functions. As a result, PU hydrolysis must be performed using a strong acid, base, and quaternary compounds that can be added to generate the active hydrogen that contains polyethers and polyamines [174]. The yield of toluenediamine vs. time shows the presence of a parallel first-order reaction equation, in which the urethane chain reacted up to 50 times quicker than urea. Urethane bonds were broken by direct hydrolysis, whereas urea bonds were broken via thermal fragmentation, to parent isocyanate and amine [175]. The recycling of PU waste with 91% of I-PU and 98% of H-PU was hydrolyzed successfully by Motokucho et al. The operating conditions of this process, the CO_2_ pressure and temperature of reaction, were up to 8.0 MPa and 190 °C, respectively. The water-soluble components were evaporated, to isolate the final products with a high yield [176]. The major drawback of the hydrolysis process is that it consumes a lot of energy to heat the batch and provide high pressure in the reactor, resulting in an uneconomical process. As a result, hydrolysis has yet to be commercialized [177,178].

Currently, glycolysis seems the most popular approach for chemical recycling of flexible and stiff polyurethane. Glycolysis is conducted by a transesterification reaction, where the hydroxyl group from glycol substitutes the ester group, by containing a carbonyl carbon of the urethane bond [179]. The common glycols applied in glycolysis are polyethylene glycol, diethylene glycol, and ethylene glycol, which are at high temperatures. In addition, the reactant and solvent should be glycol, and it is necessary to maintain the optimal ratio of PUs to glycol. Due to the long procedure time, a mixture of layers can be obtained. In addition, the recovered polyol is usually found in the top layer. It can also be used to produce new materials for PUs [180,181].

Furthermore, organometals, hydroxides, and acetates are various catalysts applied in the glycolysis of PU waste. The glycolysis can be performed with a co-reagent such as diethanol amine or amine. A reaction temperature below 180 °C results in a lack of catalytic activity, whereas a temperature above 220 °C results in unwanted amine side reactions. The major disadvantage of the chemically utilized reagents is implementing amine as a co-reactant, which causes issues with the recovered materials and is final-processed. Amines function as catalysts and accelerate the reaction between polyol and polyisocyanate by enhancing the electrophilicity of the isocyanate functional group. They also facilitate the polyols in generating PUs under uncontrolled and short-term conditions [175]. Ethylene glycol and diethylene glycol as reagents provided the best quality regarding the recovery of polyol, viscosity, and reaction time. Metal salts were used as catalysts, and lithium and Zn (Ac)_2_ showed an excellent catalytic activity, by obtaining highly pure polyol and with a short reaction time. For instance, the alkaline metal hydroxides, KOH, appeared to dissolve stiff PUs more successfully than the other catalysts [181].

An alcoholysis reaction can be conducted by combining a sequence of metal hydroxide and alcohols, such as potassium hydroxide and sodium hydroxides, under a high pressure and temperature. The reaction of alcoholysis is identical to hydrolysis reactions. Polyols and urethane compounds are produced in this process. Furthermore, to convert the waste of PUs foam into liquid, solid, and gas products, the alcoholysis process requires a high temperature and oxygen-free environment [182]. Alcoholysis with 1,2-propanediol was used to recycle polyurethane foam waste. The compounds polyol and amine were discovered [183]. Gu et al. studied the alcoholysis agent with propylene glycol and ethylene glycol, while a catalyst was applied, such as Zn_3_[Co(CN)_6_]_2_ (DMC) and KOH for PU rigid foam waste. A DMC catalyst was more effective than a KOH catalyst, according to the results of the experiments. The alcoholysis product using DMC had a higher hydroxyl content and lower viscosity, which is ideal for regenerating PU rigid foam [184].

For PU materials, gasification is a fascinating thermo-chemical feedstock recycling technique [185]. The gasification of plastic waste can be produce hydrogen compounds, generating a lot of attention for hydrogen as a future energy resource. Plastic gasification produces a variety of hydrocarbons. Nevertheless, a two-stage pyrolysis gasification method has been demonstrated to produce high-yield hydrogen from plastics. Furthermore, the pyrolysis of plastic waste is followed by steam gasification of the product in the presence of a catalyst, to produce hydrogen in a two-stage pyrolysis gasification reaction system [186]. Commercial gasification facilities have been operating for a range of feedstocks worldwide. SVZ GmbH’s pilot plant was utilized to gasify waste plastics, pelletized shredder residue, multi-solid waste, and polyurethane foam using BG-Lurgi slagging-bed gasification under a high-temperature operation [185]. Guo et al. studied the gasification and pyrolysis of waste rigid polyurethane foam (WRPUF) using a fixed-bed reactor. The yield of gaseous products from WRPUF gasification was significantly greater than that from pyrolysis. The gasification yielded more volatile nitrogen than pyrolysis. The final product of the catalytic pyrolysis and gasification of WRPUF was dramatically influenced by metallic and metal compounds as catalysts [187].

### 2.6. Recycling of Polyethylene Terephthalate

The chemical recycling of PET was reviewed, such as pyrolysis, hydrolysis, methanolysis, glycolysis, ionic liquid, phase-transfer catalysis, and combinations of glycolysis and hydrolysis, glycolysis and methanolysis, and methanolysis and hydrolysis in our previous study [20]. Furthermore, reaction kinetics and conditions were investigated theoretically and experimentally. The recycling of PET is used to solve environmental problems and find another source of raw materials for petrochemical products and energy [20]. The hydrocracking of waste plastic was pyrolyzed into high-quality liquid fuel using various catalysts, e.g., zeolite [188]. On the other hand, PET can be reprocessed by mechanical recycling to develop wooden construction bricks for building purposes, with a composition of 75 wt% wood fiber and 25 wt% plastic waste, with a total hardness up to 21.270 HRR [189]. Therefore, the mechanical recycling of plastics can be applied in large-scale industries, to solve environmental issues [190].

## 3. Down Stream Problem

Decolorization technology is a critical problem for high-quality chemical recycling and recovery of plastic wastes. Only 1% of textile wastes, mainly whites, are recycled, so the final color quality of regenerated fibers is uncontrollable. Color removal is required for large-scale circulation of non-thermoplastic fibers. Technologies for color removal from plastic wastes include dye destruction or extraction for the pre-recycling process. Mu and Yang studied the minimization of fiber density using various solvents and temperatures, completely removing dispersed dyes, acid dyes, and direct dyes from PET, nylon, and cotton fibers [191]. The plastic waste was HDPE with blue and orange colorants (pigment and/or dye). Ferreira et al. reported that biosolvents derived from renewable sources were shown to be efficient in removing colorants from HDPE packaging waste by solvent extraction (dissolution-precipitation) [192].

Plastic waste must be subjected to a series of steps (washing, grinding, density separation, sensors-driven exclusion) to eliminate further contaminants (textiles, food, glass, other polymers). The sorting step is crucial to reduce contaminants and ensure that the contaminant threshold is not reached [193]. Increased recycling rates are a proposed solution to the current health and environmental crisis that is caused by the massive overproduction of plastics. However, almost all plastics contain toxic chemicals that are not removed during recycling, but which are carried over to the new products, and the recycling process can even generate new toxic substances such as dioxins. The increased recycling is intended to contribute to a so-called circular economy, but plastics containing toxic chemicals should not be recycled. Instead, they should be considered non-circular materials. Brosché et al. reported on the increasing amount of information about toxic chemicals transferred from plastic waste into recycled plastic pellets globally [194].

The life cycle assessment (LCA) methodology and applications have been developed, and LCA continues to be an important tool for understanding the environmental impacts of materials and processes. LCA of chemical recycling processes is a growing area. A critical analysis of nine chemical recycling LCA papers found that there are two approaches to modeling: a comparison of chemical recycling methods to other plastic waste management techniques, for example, mechanical recycling or the modeling of chemical recycling methods in combination with other plastic waste management methods, to treat mixed plastic waste [15]. Marson et al. presented a life cycle assessment of PUs foams with different recycled polyol contents [195].

## 4. Conclusions

The increase of plastic usage as packaging results in more waste in the environment, resulting in significant waste pollution issues. Among plastic waste management methods, plastic recycling offers a promising approach to reducing plastic waste, while maintaining sustainability. There are various plastic recycling technologies, each of which offers various advantages and disadvantages to the user and depending on the type of plastic waste. Chemical and mechanical recycling is the typical approach to recycling different plastic materials, such as PP, PS, PVC, HDPE, LDPE, and PUs. Mechanical recycling is a convenient technique to preserve the intrinsic value of plastic, while avoiding the waste of nonrenewable resources. Chemical recycling relies on the degradation of the polymer chains to obtain a low degree of pollution. The chemical and mechanical recycling approaches facilitate plastic waste recycling with a simple process, a low cost, environmentally friendly process, and potential profitability. However, plastic waste separation before the recycling process becomes a challenge, due to the different physical and chemical characteristics of plastic waste. Since each plastic type has various properties, such as melting temperature, density, and hardness, mixed polymers do not allow retaining their original properties and practical usefulness. Therefore, to increase the value and recycling rate of plastic waste, a reliable and effective separation method for plastic waste separation is very important. Integrating recycling and plastic waste separation technologies would be an effective strategy to reduce the accumulation of environmental pollutants caused by plastic waste, particularly for industrial applications.

The explosion of plastic usage in many industries has led to environmental damage issues. The increased focus on the ecological consequences of human activities and the rising need for energy and resources has resulted in a new perspective on plastic waste streams. All stakeholders must achieve good sustainable waste management practices, to maintain sustainability. Better knowledge of plastic waste recycling will lead the policy-makers to make proper rules to overcome the environmental problems caused by plastic waste. This review presents the plastic waste recycling approaches for the properties of each material, as waste management from an environmental sustainability perspective.

## Figures and Tables

**Table 1:** The advantages and drawbacks of various methods of recycling plastic waste.

Methods	Advantages	Limitations	Ref.
Mechanical	- It is the easiest process for recycling metal matrix composites, and it is especially well-suited for fiber-reinforced polymers (FRP), where fiber breaking is accomplished through shredding.	- The decreased melt viscosity due to hydrolytic and thermal depolymerization.	[31,32]
	- The recycling facilities are simple and economical, and they use less energy and resources than chemical or physical recycling processes.	- The generation of cyclic and linear oligomers affects the printability and dyeability of the final product.	[32]
Chemical	- The greater rates of the monomer with a shorter reaction time.	- The expensive investment in developing technical infrastructure/processes.	[16,33]
	- Higher potential for profitability through new materials application.	- The feasibility of an industrial scale has not yet been completely established.	[34,35]
	- The most cost-effective approaches for high-performance recycling composites.	- High temperature and much energy are needed.	[36,37]
Biological	- The procedure is easy to follow and is also environmentally friendly.	- The depolymerization is extremely slow for the high molecular weight of hydrophobic plastic polymers.	[5]
	- Cost-effective process.		

**Table 3:** The products distribution catalytic and co-pyrolysis of PP.

Feedstocks	Catalyst	Condition			Yield of Product, %			Calorific Values of Liquid Product, kJ/kg	Major Product	Ref.
		Reactor	T, °C	t, min	Solid	Liquid	Gas			
PP	Spent FCC	Quartz Tube	510	60	NA	62	38	NA	Olefins, alkane	[86]
PP	Calcium bentonite clay	Batch	500	NA	0	88.5	~11.5	44,370	Alkene	[87]
PP + Lignocellulosic biomass; (1:2)	Spent FCC	Quartz Tube	510	60	12	52	36	NA	Aromatics, olefins, alkanes, oxygenates	[86]
PP	Bentonite clay	Fixed bed	500	10	NA	90.5	NA	44,763	Aromatics, alkanes, alkenes	[88]
PP	FCC	Stirred semi-batch	450	NA	3.6	92.3	4.1	NA	Olefins, paraffins, naphthene, aromatics	[89]
PP	Fe-SBA-15	Batch	540	300	2–0.8	73–77	24–21	NA	CH_4_, C_2_H_6_, C_3_H_6_, and C_4_	[90]
PP	Spent FCC	Batch	300	NA	2.3	72.4	23.7	43,435	Paraffin, olefins, naphthene, aromatic	[91]
pp	10% dolomite	Batch	400–500	90	NA	85.2	NA	43,000–46,000	Alkanes, alkenes	[92]
PP	Spent FCC	Stirred semi-batch reactor	400	NA	2	85	13	NA	Olefin, paraffin, naphthene, aromatic	[93]
PP	USY	Batch	450	45	1.2	82	16.8	NA	C_9_, C_12_, C_15_, C_18_ and C_21_	[94]
PP	Sulfated zirconium hydroxide	Batch	500	NA	<1	84.1	15	193.8	Paraffin, olefins	[95]
PP	Kaolin clay	Batch	450	30	23.67	67.5	8.85	46,470	Aromatics, olefins, amines, sulfide, hydroxyl	[96]

**Table 4:** Product distribution of HDPE and LDPE after catalytic pyrolysis.

Feedstock	Catalyst	Condition Operation			Yield of Product, %			Major Product	Ref.
		Reactor	T, °C	t, min	Solid	Liquid	Gas		
HDPE	HUSY	Batch	550	NA	1.9	41	39.5	C_3_–C_7_ Hydrocarbons	[165]
LDPE	Sulfated zirconium hydroxide	Batch	500	70	2	82	16	C_10_–C_24_ hydrocarbons	[95]
HDPE	HZSM-5	Batch	550	NA	0.7	17.3	72.6	C_3_–C_6_ Hydrocarbons	[165]
LDPE	HUSY	Batch	550	NA	1.9	61.6	34.5	C_4_–C_9_ hydrocarbons	[165]
HDPE	Conventional Beta zeolite	Batch	380	120	45.7	45	9.3	C_1_–C_4_; C_5_–C_12_; >C_13_ hydrocarbon	[166]
HDPE	Hierarchical Beta (CTAB)	Batch	380	120	32.7	50.3	17	C_1_–C_4_; C_5_–C_12_; >C_13_ hydrocarbon	[166]
HDPE	Hierarchical Beta (PHAPTMS)	Batch	380	120	3	81.9	15.1	C_1_–C_4_; C_5_–C_12_; >C_13_ hydrocarbon	[166]
LDPE	HZSM-5	Batch	550	NA	0.5	18.3	70.7	C_3_–C_7_ hydrocarbons	[165]
LDPE	Bentonite	Fixed bed	700	NA	NA	86.6	NA	C_5_–C_9_; C_10_–C_13_; >C_13_	[88]
HDPE	Bentonite	Fixed bed	700	NA	NA	88.7	NA	C_5_–C_9_; C_10_–C_13_; >C_13_	[88]
HDPE	Sulfated zirconium hydroxide	Batch	500	70	<1	79.5	20.1	C_10_–C_24_ hydrocarbons	[95]
HDPE	FCC	Semi-batch	420	60	4.2	89.1	6.7	C_4_–C_9_ Hydrocarbons	[167]
HDPE	MFI Zeolite—Syn	Flask	380	60	-	51	49	C_5_–C_7_ hydrocarbons	[168]
HDPE	Silica/NaOH	Packed bed	500	70	-	82	18	C_10_–C_28_ hydrocarbons	[169]

## Data Availability

No applicable.

## Abbreviations

ABS	Acrylonitrile butadiene styrene
EG	Ethylene glycol
FRP	Fiber-reinforced polymers
HDPE	High-density polyethylene
HRR	Rockwell R-scale hardness
LDA	Linear discrimination analysis
LDPE	Low-density polyethylene
LIBS	Laser-induced breakdown spectroscopy
PA	Polycaprolactam
PAHs	Polycyclic aromatic hydrocarbons
PBX	Plastic bonded explosive
PC	Polycarbonate
PCA-SVM	Principal component analysis
PE	Polyethylene
PLS	Partial least squares regression
PLS-DA	Partial least squares discrimination analysis
PET	Polyethylene terephthalate
PMMA	Poly(methyl methacrylate)
PP	Polypropylene
PVC	Polyvinyl chloride
PS	Polystyrene
PU	polyurethane
SERS	Surface-enhanced Raman spectroscopy
